# Coarse-grained computer simulation of dynamics in thylakoid membranes: methods and opportunities

**DOI:** 10.3389/fpls.2013.00555

**Published:** 2014-01-21

**Authors:** Anna R. Schneider, Phillip L. Geissler

**Affiliations:** ^1^Biophysics Graduate Group, University of CaliforniaBerkeley, CA, USA; ^2^Department of Chemistry, University of CaliforniaBerkeley, CA, USA; ^3^Chemical Sciences and Physical Biosciences Divisions, Lawrence Berkeley National LaboratoryBerkeley, CA, USA

**Keywords:** simulation, coarse-grained modeling, nanoscale, thylakoid membrane, photosystem, Monte Carlo, Brownian dynamics

## Abstract

Coarse-grained simulation is a powerful and well-established suite of computational methods for studying structure and dynamics in nanoscale biophysical systems. As our understanding of the plant photosynthetic apparatus has become increasingly nuanced, opportunities have arisen for coarse-grained simulation to complement experiment by testing hypotheses and making predictions. Here, we give an overview of best practices in coarse-grained simulation, with a focus on techniques and results that are applicable to the plant thylakoid membrane–protein system. We also discuss current research topics for which coarse-grained simulation has the potential to play a key role in advancing the field.

## 1. Introduction

Photosynthesis spans disparate length and time scales, from femtosecond quantum events to seasonal canopy-scale processes. Many of the key phenomena that regulate the efficiency of light-harvesting and charge separation in the thylakoid membrane occur on intermediate scales—nanometers to microns in space, and milliseconds to minutes in time. In this regime, it has been experimentally challenging to simultaneously probe structure and function. On one hand, atomic force microscopy (AFM) and electron microscopy (EM) and tomography (ET) have proven to be powerful tools for elucidating the organization of the thylakoid membrane architecture and of its intrinsic pigment-proteins, but they typically lack sufficient temporal resolution to resolve dynamic structural fluctuations in detail. On the other hand, the popular biochemical, spectroscopic, and fluorescence microscopy methods are effective for exploring protein behavior within and trafficking between grana and stroma lamellae, but obscure functional distinctions between the many local environments within each membrane region. In addition, while isolated grana are useful model systems, the complexities of thylakoid morphology and pigment-protein synthesis have made it difficult to construct minimal reconstituted systems that would enhance experimental control over the membrane's structure and contents.

Coarse-grained computer simulation has the potential to bridge the gap between these experimental techniques. Computer simulation offers control over every input to the system (e.g., the number and identity of particles), and access to every output (e.g., particle locations over time), enabling the researcher to test hypotheses and make predictions about relationships between thermodynamic parameters and biologically-relevant phenotypes. Here, we specifically consider topics that fall within the purview of soft mattter biophysics, by focusing on fluctuating nanoscale properties of the biomolecules and biomaterials that form the milieu for photosynthetic function. Coarse-grained modeling is powerful and widely applicable, yet, like any scientific technique, requires attention to a host of subtleties if it is to yield meaningful insights (Frenkel, 2013).

In this Mini Review, we outline key considerations for building and utilizing coarse-grained models of plant photosynthetic membrane systems, and give examples of research topics in this field where simulation has been or could be a valuable complement to experiment.

## 2. Modeling approaches

### 2.1. Energetics

Coarse-grained models derive their strength from the clarity and broad applicability of their assumptions, rather than from the precision of their details. Therefore, the potential energy function governing a coarse-grained model should include the simplest set of particles, interactions, and material properties that captures the phenomenology of interest and is motivated by the underlying physics.

For instance, consider the nanoscale properties of a generic protein: it occupies space; it may have a compact shape; it may form specific contacts with other proteins; it may aggregate or crystallize. In many cases, these properties can be represented mathematically by pairwise energetic potentials between protein particles.

The basic features of appropriate potentials between particles representing proteins (or strongly bound complexes) are often clearly dictated by their scale and molecular nature. For example, forces between proteins with well-defined internal structure should include a steric contribution, establishing the volumes they occupy. This repulsive interaction is harsh, acting over short range, and can be reasonably caricatured as a singular “hard core.” Continuous potentials that achieve the same effect, such as the Weeks-Chandler-Andersen (WCA) potential (Weeks et al., 1971), are sometimes preferred for practical reasons. Purely repulsive hard spheres were among the earliest model systems studied with molecular simulation techniques and, despite their simplicity, display a rich phase behavior (Alder and Wainwright, 1957). Phase diagrams have also been explored for non-spherical hard shapes, including rods (Bolhuis and Frenkel, 1997), ellipsoids (Odriozola, 2012), cubes (Smallenburg et al., 2012), and exotic polyhedra (Damasceno et al., 2012; Henzie et al., 2012).

Cohesion between proteins in solution can emerge from many sources, e.g., hydrogen bonding, screened Coulomb interactions, hydrophobic effects, and other solvent-mediated effects. Despite this variety, however, the attractions effected by these forces are similar in nature at the coarse-grained level. In particular, they typically attenuate over distances that are short compared to the scale of proteins themselves. They thus act primarily as contact potentials, which reward close approach of coarse-grained particles. A minimal model of this behavior is a “square-well” potential that has a constant strength within a cutoff radius and is non-interacting beyond the cutoff radius. Other models use the Lennard-Jones (LJ) potential to accurately capture the *r*^1/6^ decay of van der Waals interactions (Lennard-Jones, 1924), the Yukawa potential to capture screened electrostatics (El Mendoub et al., 2010), or custom functional forms to capture other phenomenology [e.g., (Pasqua et al., 2010; Schneider and Geissler, 2013)]. Square-well and Yukawa potentials are typically used in concert with a hard-core repulsion; the LJ potential includes its own volume exclusion term, from which the WCA potential is derived. Any attractive interaction can be made to act isotropically, favoring non-specific aggregation, or between interaction sites on so-called “patchy” particles, often favoring self-assembly of specific structures (Haxton and Whitelam, 2012).

Lipid bilayer membranes are modeled in different ways depending on the desired range of bending fluctuations. If the membrane is essentially flat and bending fluctuations are not expected to affect the phenomenon of interest, it can be modeled simply as a static planar surface through which particles representing intrinsic membrane proteins can travel. Because grana lamellae appear flat in most electron micrograms and tomograms [reviewed in Dekker and Boekema (2005); Daum and Kühlbrandt (2011); Nevo et al. (2012)], this approach has been used for coarse-grained simulations of grana proteins (Drepper et al., 1993; Tremmel et al., 2003, 2005; Kirchhoff et al., 2004; Schneider and Geissler, 2013).

In more generality, a membrane can be well described by the Helfrich Hamiltonian for an elastic sheet (Safran, 2003). When only small-amplitude fluctuations about a planar equilibrium state are considered, it is convenient to represent the membrane in the Monge gauge [i.e., each point (x,y) in the plane is associated with a height *h*(x,y) above the plane] and linearize the Hamiltonian to decouple the Fourier modes (Safran, 2003). If significant changes in membrane curvature or topology are essential to the research question, then the membrane can be represented as a triangulated set of tethers and nodes (Gompper and Kroll, 1997), or as a collection of “membrane patch” particles (Pasqua et al., 2010). To represent the internal structure of lipids at higher resolution, various models have been developed [e.g., (Izvekov and Voth, 2009; West et al., 2009; de Meyer et al., 2010)].

### 2.2. Dynamics

Coarse-grained biophysical models forego an explicit solvent for computational and conceptual efficiency. They instead include the random buffeting of an implicit solvent via stochasticity and/or friction in the algorithm that generates new configurations in a trajectory. Two common classes of algorithms for this purpose are Langevin dynamics and Metropolis Monte Carlo, which we sketch here; see the excellent textbooks (Allen and Tildesley, 1989; Frenkel and Smit, 2001) for thorough explanations and implementation guidelines.

Overdamped Langevin dynamics, also known as Brownian dynamics, propagates a system by integrating equations of motion that include deterministic forces derived from the potential energy function, as well as random forces parameterized by diffusion coefficients. For systems of particles, the integration is typically performed in real space; for a membrane in the Monge gauge, it is often easier to perform the integration in Fourier space (Brown, 2008). Because the equations of motion involve gradients of the potential, standard algorithms for Brownian dynamics require the interaction energies to be differentiable.

Metropolis Monte Carlo takes a conceptually different approach: each new configuration is generated from the previous one by proposing a random perturbation or “move” of the system, then accepting or rejecting the move according to the Metropolis-Hastings acceptance criterion. The researcher is permitted considerable leeway in selecting the types of moves that are proposed. These moves can be physically motivated, such as small displacements or rotations of single particles, or they can be starkly non-physical, such as the insertion or deletion of entire particles; in either case, moves should be designed to efficiently sample the most important regions of state space. The main requirement is that the proposal–acceptance scheme must obey detailed balance so that the process creates a Markov chain with a well-defined stationary distribution. Advanced Monte Carlo methods include algorithms for free energy calculations [e.g., umbrella sampling with MBAR (Shirts and Chodera, 2008)], equilibration on rough landscapes [e.g., replica exchange (Earl and Deem, 2005)], and rare event sampling [e.g., transition path sampling (Bolhuis et al., 2002)].

The choice of simulation dynamics algorithm often hinges on whether it is more important to fully characterize the system's equilibrium properties, or to most realistically capture its dynamics. Brownian dynamics and Monte Carlo dynamics can both accurately simulate time correlations in the low-Reynolds-number systems of biology in some limits, although hydrodynamic effects can be difficult to compute correctly (Ermak and McCammon, 1978; Berthier and Kob, 2007; Brown, 2008); both can require large computational resources to explore the system's equilibrium fluctuations. Cleverly-chosen Monte Carlo moves can dramatically reduce the computational time necessary to sample the equilibrium distribution, yet these moves are often ones that create highly unphysical dynamics. However, compromises exist; for instance, virtual-move Monte Carlo is a computationally-efficient Monte Carlo method that yields the correct dynamic behavior both for single particles undergoing free diffusion, and for large clusters undergoing collective motion (Whitelam and Geissler, 2007).

### 2.3. Emergent behavior

In equilibrium simulations with Monte Carlo or Brownian dynamics, as in reality, entropy and energy together determine which states are most probable. The complex interplay between energetic and entropic forces can give rise to striking self-assembled structures and counterintuitive collective phenomena, even in seemingly simple systems. Discovery, characterization, and prediction of such emergent properties are frequent goals of coarse-grained modeling.

Several classes of entropic forces are likely to be important in photosynthetic protein–membrane systems. One is depletion-attraction, an effective attraction that brings some components of a system closer together in order to maximize the entropy of other (often smaller) components (Asakura and Oosawa, 1958). Depletion-attraction is ubiquitous in biological systems (Marenduzzo et al., 2006), and it is the driving force behind the crystallization of hard particles (described above). Another entropic force acts between layers of a stack of membranes, whose out-of-plane fluctuations are suppressed by steric constraints imposed by neighboring layers Helfrich (1978). An accurate accounting of this force is necessary to understand the adhesion of membrane stacks (Lipowsky and Leibler, 1986).

Other emergent behaviors can arise from coupling between membranes and membrane proteins. For instance, membrane-mediated forces between intrinsic membrane proteins are caused by hydrophobic mismatch (Harroun et al., 1999; Schmidt et al., 2008), membrane curvature (Tian and Baumgart, 2009), and lipid composition (de Meyer et al., 2010). Conversely, membrane-associated proteins can sculpt the membrane's morphology and composition fluctuations (McMahon and Gallop, 2005; Stachowiak et al., 2012).

Phase transitions are the paragon of collective behavior in statistical mechanics. Characterized by non-analytic change (e.g., a discontinuity) in an observable quantity, they arise not only in simple molecular substances (e.g., water freezing or boiling) but also in a variety of biophysical contexts, including membrane binding-unbinding transitions (Lipowsky and Leibler, 1986) and protein crystallization (Schneider and Geissler, 2013). At phase coexistence, a system can stably exist in each of two very different states, but is unstable as a mixture of the two (unless coexistence is allowed by the thermodynamic ensemble) (Chandler, 1987). Even if only one phase is stable at a time, the kinetics of phase transitions can nevertheless be complex. In classical nucleation theory, it is first slow to overcome the free energy barrier to nucleation of the new phase inside the old, then fast for nuclei to begin to grow, and finally (for high-symmetry phases) slow for defects to anneal; even more sluggish and exotic dynamics can be observed in practice (Whitelam et al., 2009; Hedges and Whitelam, 2011).

## 3. Applications

### 3.1. Membrane morphology

The mechanism of membrane curvature generation in the photosynthetic purple bacterium *Rhodobacter sphaeroides* has been investigated by Monte Carlo simulation (Frese et al., 2008). In that work, four physical features of the biological system were included in the coarse-grained model: the flexibility and fluidity of the membrane, the high packing fraction of reaction center-light harvesting 1 (RC-LH1) complexes and light harvesting 2 (LH2) complexes in the membrane, the size disparity between RC-LH1 and LH2 complexes, and the wedge shape of the complexes. Thus, the coarse-grained model consisted of a fluctuating triangulated network representing the membrane, with hard spheres at the vertices representing protein complexes. By varying the spontaneous curvatures and diameters of the hard sphere vertices in equilibrium simulations, the authors found support for the hypotheses that protein shape (via the Helfrich energy) and crowding (via depletion-attraction) both influence the organization of the bacterial photosynthetic membrane.

Similar approaches could be used to understand the morphogenesis of the plant thylakoid membrane. There is experimental evidence that crowding in the aqueous stroma induces an entropic attraction between membrane layers in grana stacks (Kim et al., 2005). This hypothesis could be tested by constructing a triangulated or membrane-patch-model membrane with a thylakoid-like topology, equilibrating the membrane in the presence of varying densities of hard spheres in the stroma-like space, and measuring grana cohesion. In addition, the influence of energetic forces that have been proposed to play important roles in grana formation (Chow et al., 2005; Schneider and Geissler, 2013) could be investigated by introducing appropriate coarse-grained potentials between fluctuating membrane sites. Independent control over each driving force has not been achieved in experiment, but could be straight-forward in simulation, allowing computational studies to advance our understanding of the biophysics of thylakoid morphology.

### 3.2. Protein self-assembly

Thylakoid membrane proteins, particularly photosystem II (PSII) and light-harvesting complex II (LHCII), display a variety of self-assembled structural motifs [reviewed in Dekker and Boekema (2005); Kouil et al. (2012); Nevo et al. (2012)]. Kirchhoff, Tremmel, and coworkers investigated crowding effects on PSII and LHCII organization by conducting Monte Carlo simulations of a coarse-grained model in which the protein complexes were represented by hard particles with structurally-detailed shapes, and the membrane was represented by a discretized static 2d sheet (Kirchhoff et al., 2004; Tremmel et al., 2005). This approach was computationally challenging because the discretization of space introduced artifacts when a short-range attraction was added to the model, and may have exacerbated the inherent difficulty of equilibrating a dense system using single-particle Monte Carlo moves (Tremmel et al., 2005). These studies concluded that a richer model would be necessary to capture key structural motifs observed *in vivo* (Kirchhoff et al., 2004).

**Figure 1:**
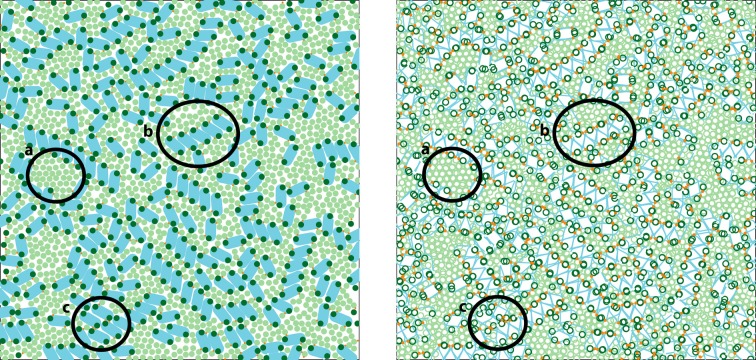
**Snapshot from a simulation of the coarse-grained model described in Schneider and Geissler (2013).** The **left** panel shows the top (stroma-side-up) layer of the two-layer geometry, and the **right** panel shows an overlay of the particles in both layers. The system size is comparable to a granum *in vivo*: each square simulation box has a side length of 400 nm and contains LHCII (green) and PSII (blue) particles at a total packing fraction of 0.75, with a stoichiometry of free LHCII trimers to PSII C_2_S_2_ super- complexes of 6. Various self-assembled structural motifs are highlighted: (a) entropic ordering of LHCII particles; (b) stacking-mediated linear aggregates of PSII particles; (c) precritical nucleus of PSII C_2_S_2_ crystal.

To explore the role of LHCII intermembrane stacking on PSII-LHCII organization, we extended the Kirchhoff-Tremmel model by introducing a second membrane layer that is coupled to the first layer via a phenomenological potential that mimics stroma-side stacking interactions between LHCII complexes (Schneider and Geissler, 2013). In addition, our model used simplified particle shapes and intramembrane interactions, which had the benefits of increasing the model's generality, obviating the need for discretized space, and permitting direct comparison to well-characterized models of hard discs and discocylinders. Monte Carlo simulations of this model found that LHCII stacking interactions were necessary to recapitulate PSII crystalline arrays, a widely observed yet poorly understood structural feature of grana (Dekker and Boekema, 2005; Kouil et al., 2012; Nevo et al., 2012), as well as other structural motifs (Figure 1). Furthermore, free energy calculations in the appropriate thermodynamic ensemble mapped the phase boundaries between regions of physiologically-relevant parameter space that favor purely ordered crystal, purely disordered fluid, or coexisting crystal and fluid phases of PSII-LHCII mixtures.

This model could be adapted to investigate the factors that stabilize other grana structural motifs. For instance, LHCII compelexes appear to aggregate under conditions that create the qE component of non-photochemical quenching (Johnson et al., 2011; Ruban et al., 2012). Many of the proposed physicochemical processes associated with qE could favor LHCII aggregation: increased protonation or conversion of bound violaxanthin to zeaxanthin (Ruban et al., 2012) could, either directly or indirectly (e.g., by inducing a conformational change of LHCII), increase energetic attractions among LHCII complexes to favor LHCII clustering, or could decrease energetic attractions between LHCII and PSII complexes to favor LHCII phase separation from PSII. On the other hand, a qE-associated conformational change of LHCII that decreases its volume (van Oort et al., 2007) would decrease the entropic driving force for aggregation. Equilibrium simulations of coarse-grained models with additional specific (i.e., patchy) or non-specific (i.e., isotropic) interactions may be able to distinguish between these hypotheses and lead to predictions that could be confirmed by experiments.

### 3.3. Mobility

Particle mobility due to Brownian motion is characterized by a diffusion coefficient and can be studied experimentally by techniques like fluorescence recovery after photobleaching (FRAP) (Kirchhoff et al., 2008, 2013; Goral et al., 2010; Johnson et al., 2011; Herbstová et al., 2012) or single-particle tracking (Consoli et al., 2005). After a perturbation that brings a system out of equilibrium, diffusion leads to fluxes of particle density that act to smooth out gradients in chemical potential, whether or not the perturbation changed the intrinsic particle diffusion coefficients. In contrast, in an equilibrium system, Brownian motion does not lead to net transport of particles, even if diffusion coefficients are large.

Plastoquinone mobility was considered using simulations of the Kirchhoff-Tremmel model, yielding a percolation threshold for plastoquinone at 60–70% protein packing fraction (Tremmel et al., 2003, 2005). LHCII diffusive transport from grana to stroma lamellae during state transitions was studied via Monte Carlo simulation of a mixture of hard discs; by fitting to time series of membrane fractionation data, the authors concluded that phosphorylated LHCII has a higher diffusion coefficient than unphosphorylated LHCII (Drepper et al., 1993). In the future, coarse-grained models in which desired self-assembly properties have been demonstrated could be used to explain or predict the dynamics of protein mobility that accompany perturbative phenomena like qE, state transitions, and PSII photoinhibition and repair.

## 4. Outlook

Recent advances in experimental techniques for thylakoid biophysics could aid in the parameterization and verification of coarse-grained models. Reconstituted proteoliposomes like the one demonstrated in (Wilk et al., 2013) allow greater control over the identity and quantity of each component of the system, creating experimental model systems that are more directly comparable to simulation models. In addition, AFM methods including high-speed AFM (Casuso et al., 2010) and force-spectroscopy AFM (Liu et al., 2011) have been used to determine protein interaction free energies and diffusion coefficients in bacterial photosynthetic membranes; similar studies of the plant photosynthetic apparatus could inform and test coarse-grained models.

In summary, the field of coarse-grained modeling is well-developed and has much to offer to the molecular photosynthesis community, although care must be taken when constructing, simulating, and interpreting the results of such models. Coarse-grained simulation and experiment have great potential to play complementary roles in future studies of dynamic nanoscale processes in plant photosynthetic membranes.

### Conflict of interest statement

The authors declare that the research was conducted in the absence of any commercial or financial relationships that could be construed as a potential conflict of interest.
